# The impact of armed conflict on adolescent transitions: a systematic review of quantitative research on age of sexual debut, first marriage and first birth in young women under the age of 20 years

**DOI:** 10.1186/s12889-016-2868-5

**Published:** 2016-03-04

**Authors:** Sarah Neal, Nicole Stone, Roger Ingham

**Affiliations:** Department of Social Statistics and Demography, Social Sciences, University of Southampton, Southampton, S017 1BJ UK; Centre for Sexual Health Research, Department of Psychology, University of Southampton, Southampton, S017 1BJ UK

**Keywords:** Young women, Armed conflict, Sexual health, Early marriage, Fertility

## Abstract

**Background:**

Young women in conflict-affected regions are at risk of a number of adverse outcomes as a result of violence, economic deterioration and the breakdown of community structures and services. This paper presents the findings of a systematic review of quantitative literature reporting how key sexual and reproductive health (SRH) outcomes among young women under the age of 20 years are affected by exposure to armed conflict; namely, sexual debut, first marriage and first birth. Increases in these outcomes among young women are all associated with potential negative public health consequences. It also examines and documents possible causal pathways for any changes seen.

**Methods:**

To fit with our inclusion criteria, all reviewed studies included outcomes for comparable populations not exposed to conflict either temporally or spatially. A total of 19 studies with results from 21 countries or territories met our inclusion criteria; seven presented findings on marriage, four on fertility and eight on both of these outcomes. Only one study reporting on sexual debut met our criteria.

**Results:**

Findings show clear evidence of both declines and increases in marriage and childbirth among young women in a range of conflict-affected settings. Several studies that showed increases in marriage below the age of 20 years reported that such increases were concentrated in the younger teenagers. Trends in fertility were predominantly driven by marriage patterns. Suggested causal pathways for the changes observed could be grouped into three categories: involuntary, gender and psycho-social and economic and material factors.

**Conclusion:**

The review reveals a paucity of literature on the impact of conflict on SRH outcomes of young women. Further quantitative and qualitative studies are needed to explore how conflict influences SRH events in young women over both the short- and longer-term.

**Electronic supplementary material:**

The online version of this article (doi:10.1186/s12889-016-2868-5) contains supplementary material, which is available to authorized users.

## Background

Young women living in conflict affected countries face risks that are likely to have negative effects on their sexual health and wellbeing. Violence, deterioration of economic conditions, poor access to services and breakdown of community norms place them at risk of sexual violence, forced marriage, transactional and/or coerced sex, and other risky sexual behaviours. However, quantitative evidence is rarely presented to support these reports of increased risks, and much of the increasing awareness of how conflict impacts on the reproductive health of girls and young women is derived from relatively small scale qualitative data, including case studies [1–3]. These studies provide highly valuable individual-level insights but cannot assess the scale of the impact at either a population level or for vulnerable sub-groups. For example, many qualitative reports and media coverage suggests that conflict increases the incidence of child marriage, but there is little evidence from quantitative studies.

In order to explore this issue we carried out of a systematic review of quantitative literature that reported how three key sexual and reproductive health (SRH) outcomes among young women under the age of 20 years - sexual debut, first marriage and first birth - are affected by exposure to armed conflict in a number of countries and settings. These three indicators are all known to have potential negative health and social consequences when they occur at an early age, and thus a knowledge of whether the incidence of these events changes in this context is important for guiding programmes designed to protect and support women in conflict and post-conflict environments. The time when sexual activity commences marks the beginning of a young person’s exposure to a number of adverse reproductive outcomes; for example, early sexual debut is associated with increased risk of unintended pregnancy and sexually transmitted infections (STIs), including HIV [4, 5]. In many cultures, marriage before the 20^th^ birthday is common; while this may be viewed as a positive transition by the community and, in many cases, the young woman herself, child marriage (defined as marriage under the age of 18 [6]) is associated with a number of adverse health outcomes [7] as well as educational, and socio-economic disadvantage [8]. The increased risk of maternal mortality faced by adolescent women is probably less severe or widespread than previously believed [9], but increased risk to the infant is well documented [10]. In addition to physical and socio-economic risk, early childbearing, particularly outside marriage, may also lead to stigmatisation and isolation in some contexts [11].

As well as presenting the findings from the included studies, we explore the suggested pathways through which conflict may affect these outcomes in order to shed light on how impacts may vary within different contexts. We also discuss the quality of research in this area and suggest ways that further evidence can be developed and strengthened.

## Method

### Procedure

Our systematic review followed a defined protocol to reduce bias and ensure replicability. Initially, six electronic databases were searched: Medline/Pubmed, ASSIA/Proquest, CINAHL, EMBASE, African Index Medicus and Reproductive Health Library. As well as peer reviewed articles, our inclusion criteria allowed for theses, dissertations, reports and grey literature as long as data sources used were clearly referenced, so searches were also conducted using Zetoc and search engines such as Google. Websites of relevant United Nations and non-governmental organisations and networks were hand searched, along with references from identified papers. Studies using any quantitative methodologies were included as long as they met the other criteria, but purely qualitative studies were excluded.

Our search terms are shown in Additional file 1: Appendix 1. Only articles written in English from 1970 onwards were eligible for inclusion in the search. Our initial database and hand search yielded a total of 1298 documents once duplicates were removed. Details of all papers identified from the initial search were uploaded onto Eppi-reviewer (http://eppi.ioe.ac.uk/). Two reviewers (SN and NS) carried out initial screening of all identified studies by title and abstract. Fifty studies judged to be potentially eligible for inclusion and complete articles and reports were then retrieved.

### Inclusion of studies

These sources were reviewed independently by two authors (SN and NS) to ascertain whether they met the eligibility criteria. Any disagreement was resolved by discussion with added input as required from the third author (RI); there was an initial agreement rate of over 90 %. In cases of uncertainty, the authors of the papers were contacted for clarification.

The inclusion criteria allowed for several different measures of change of age at first sex, age at marriage and age at first birth within our age range of interest (see Table 1). Changes in median or mean age were not accepted, as these did not automatically reflect a change in age of the event for our specific age group. Studies showing change in risk or odds for an age group wider than under 20 years (e.g. 15–24) were excluded.

**Table 1:** Summary of outcome measures defined by inclusion criteria

Outcome	Measures included in study
Age at first sex	Percentages of women reporting sexual debut before the age of 20 years
	Odds ratio or relative risk of first sex by a specified age of below 20 years
Age at first marriage/union	Percentages of women reporting first marriage/union the age of 20 years
	Odds ratio or relative risk of first marriage/union by a specified age of below 20 years
Age at first birth	Age specific fertility rates 15–19 (or for other age grouping with upper limit below 20 years)
	Percentages of women reporting age at first birth by a specified age of below 20 years
	Odds ratio or relative risk of first birth by a specified age of below 20 years

Studies were only included if they reported comparable outcomes for populations identified as exposed or not exposed to a conflict that was clearly documented as active during the period of measurement in the study; databases from the Uppsala Conflict Data Program were used to clarify this (http://www.pcr.uu.se/research/UCDP/). Only conflicts after 1970 were included, as we wanted to focus on the impacts of recent conflicts and data quality from historical studies is difficult to verify. Exposure could be measured by temporal, geographical or individual level data, or be based on specific impacts of conflict on sub-populations (e.g. displacement). However, to ensure that the comparator was appropriate, studies measuring outcomes amongst displaced people were only included if the comparison population was from the same country of origin. In terms of temporal exposure, we included studies that presented findings for pre-conflict and in-conflict, and in-conflict and post-conflict. In several cases, the acute period of the conflict was too short to measure change (i.e. the Rwandan genocide) so in cases where the period of conflict was less than one year we included measurements pre- and within the first five years post-conflict.

### Data extraction and quality assessment

Data extraction was carried out using a structured format which drew on the recommendations of the STROBE guidelines for reporting observational research [12]. The information extracted included:

- country/region/date of study period;
- description of the populations studied (exposed and non-exposed);
- description of the conflict (including categories of conflict type and intensity developed by the Uppsala conflict project);
- data source and sampling;
- study design/methodology;
- method of measuring exposure to conflict; and
- results – i.e. change in outcome, and any suggested causal pathway

A checklist tool for rating quality was developed based on recommended practice [13, 14] and studies were rated for quality based on such factors as sample selection and size, measurement methods for outcome, methodology, adjustment for other confounding factors, method of measuring exposure to conflict and description of population/conflict environment. However, all studies were included regardless of their assessed quality.

An initial scoping exercise of the published research literature highlighted substantial heterogeneity in country contexts, study designs, study aims and objectives, data sources and conflict comparators utilised. Comparison and appraisal of these types of studies is invariably complex and calls for flexible use of the published guidelines on systematic reviews; it would have been inappropriate to have attempted a statistical meta-analysis. We therefore chose a narrative synthesis approach for reporting patterns of change in our chosen indicators. The heterogeneity of contexts also meant that we considered it important to not just document the change in indicator, but also the proposed pathway by which this change came about. In addition to our narrative synthesis, we conducted a combined framework and thematic synthesis of suggested possible pathways when these were included in the literature. This enabled a better understanding of the key concepts and dimensions seen in diverse contexts concerning the impact of conflict on the selected outcomes.

We based our initial analysis of causal pathways on a framework presented by Staveteig [15], which identifies four groups of factors that can influence the outcomes - involuntary factors, material and economic factors, sex and gender roles and psychosocial factors, although we later merged sex and gender roles and psychosocial factors for our analysis as many cultural factors seemed to straddle both categories. Reading the abstracts and, subsequently, the full texts of the studies allowed us to identify new and emerging themes.

## Results

### Description of the articles

We identified 21 articles, representing 19 unique studies, which presented SRH outcome data over a period of armed conflict. Studies that overlapped on country and time periods were deemed to be different if they were by different authors, used different data sources or presented markedly different findings. In two cases there were two papers each linked to one study [16–19]; since slightly different data and context are presented in these papers we cite both in relation to each study. However, we usually refer to numbers of studies rather than numbers of papers to overcome the bias related to multiple papers from the same study. A Prisma Flowchart is included as Additional file 2: Appendix 2. The articles covered 14 different settings; Table 2 provides a summary of the primary focus of the research and geographical region.

**Table 2:** Number of studies by setting and sexual health outcome (NB A number of studies provided data for two countries or territories)

	Number of studies	Sexual debut	Marriage	Fertility
Middle East				
Iran	2	-	Y	Y
Lebanon	1	-	Y	-
West Bank	4	-	Y	Y
Gaza	4		Y	Y
Syria	1	-	Y	-
Eastern Europe				
Bosnia	1	-	Y	Y
Central and Southern Asia				
Tajikistan	2*	-	Y	Y
Bangladesh	1	-	-	Y
Nepal	1	-	Y	-
Eastern Asia				
Cambodia	2	-	Y	Y
Northern Africa				
Mali	1	-	Y	Y
Eritrea	2*	-	Y	Y
Southern Africa				
Rwanda	1		Y	Y
Uganda	1	Y	-	-
*Total*	21*			

*This table refers to the number of papers rather than the number of unique studies on which they area based

The specification of age grouping within our sample group of interest (under 20 years) varied between the studies; this may reflect the status of women, the legal age of marriage and the childbearing patterns within the countries studied. The majority used 15 to 19 years; however, several studies also included data for younger women or disaggregated age groupings further (for example [16, 17, 20–25]).

As shown in Additional file 3: Appendix 3 (available online), the majority of studies presented a temporal comparison of the possible impact of conflict before and during conflict or before, during and after conflict. One study examined exposure to conflict based on the number of child abductions in Nepal [24], and a further study compared outcomes amongst young women living in displaced persons’ camps with those in surrounding villages in Uganda [23]. Data from Save the Children combined refugee status with a temporal analysis, comparing the percentages of women married before aged 20 in Syria before the war to Syrian young women now living in Jordan refugee camps [25].

All but one study used existing data sources, including Demographic and Health Survey (DHS) or World Fertility Survey (WFS), Census Data, vital registration and national living standards surveys. The remaining study collected primary data on sexual debut by use of a structured questionnaire [23]. Simple cohort and/or period trend analyses were presented in the majority of studies, using a range of outcomes including percentages of women married (or unmarried) by a certain age, and age specific fertility or marriage rates. Hazard ratio probabilities or relative risks were used in two studies [22, 24] and logistic regression was used in one paper [16].

Of the 21 papers (based on 19 studies) 12 were rated as low quality and nine were rated as moderate. Limitations are outlined more fully in the discussion; however, it must be recognised that in a number of the papers the outcome of interest to our review was not the main focus of the research, and was presented as descriptive data (sometimes as a line graph) to support a more comprehensive analysis of other outcomes or as a subsection of a wider study.

A summary of findings is provided in Additional file 4: Appendix 4 (available online).

### Sexual debut

Only one study fitted our selection criteria and described the impact of conflict on young women’s sexual debut. Okae [23] compared data on sexual debut of young people within IDP camps in the Lira region, Uganda, to those of young people in the surrounding settlements. Differences were not substantial, but were larger amongst young men than amongst young women.

### Impact of conflict on marriage

Using the themes included in the Staveteig framework, the factors proposed as being associated with either increases or declines in marriage rates could be labelled as belonging to one of the three following groupings: (i) material and economic (e.g. lack of basic resources), (ii) involuntary (e.g. displacement and mortality), and (iii) gender and psychosocial (e.g. increased pro-natalism). The majority of studies identified more than one causal factor across the groupings.

#### Increase in marriage

In six studies, an increase in marriage was reported during a period of conflict [15–17, 22, 24–26]. In some cases, these increases were quite marked; for instance, Save the Children [25] reported that the proportion of registered marriages in Jordan among Syrian under-18 refugee women had risen from 12 % in 2011 (roughly the same as the figure in pre-war Syria) to 18 % 1 year later.

A number of the pathways suggested for potential impact fall within the gender and psycho-social grouping. Clifford [22] and Shemyakina [16, 19] both reported an increase in marriage in young women aged between 15 and 17 years during the peak years of the hostilities in Tajikistan. Parental fear that a young woman’s honour could be tainted in times of conflict was reported by both authors as a key reason. Save the Children’s study in Syria [25] also suggested protection of a young woman’s honour was a driving force for increasing early marriage. In Mali, Randall [26] showed that the proportion of first marriages involving very young (below 15 years old) Tuareg girls increased during the conflict years, with levels only reverting to those seen pre-conflict after the war ended. The author concluded that early marriage provided protection for young women against premarital pregnancy. Further, the refugee camps enhanced both the desire and opportunity for improved social cohesion, and reinforced old family alliances and created new ones, resulting in increased first marriage rates among the very young. Valente [24] used regression techniques with large survey data to show that exposure to increased intensity of conflict in Nepal was associated with an increased probability of marriage before 15 years.

The study in Syria also suggested an economic element to the changes observed, arguing that many of the marriages for young women in refugees camps were deemed transactional by parents, frequently occurring with older men and seen as a way of securing financial sponsorship of their daughters [25].

Staveteig [15] analysed data from the 2000 and 2005 Rwandan DHS to demonstrate acceleration in marital timing among the birth cohort aged between 15 and 19 years at the time of the genocide. This was contrary to what had been expected given the demographic transitions occurring within the country and the practice of early marriage becoming increasingly less common. Later cohorts (post-conflict) then appeared to revert back to the expected trend of increasing age at marriage. The author suggests that the change observed may have been due to involuntary factors as young women entered the marriage market earlier than normal in response to the reduced availability of male partners. Further, many of the additional marriages were deemed more likely to be primarily transactional, following orphanhood and the destruction of homes and livelihoods.

In four studies there was a clear pattern of younger teenagers experiencing increased rates of marriage, which was not evident among older teenagers [16, 17, 22, 24, 26]. In Tajikistan, both Shemyakina [16, 17] and Clifford [22] found that marriages at the ‘peak’ age of 18 to 20 years (which takes this group out of the age range for our review) actually declined somewhat during the conflict. Randall’s study in Mali [26] failed to detect any increase in marriage for the 15 to 18 age group despite a marked increase for girls below 15 years and, in Nepal, Valente [24] did not detect an increased probability of marriage for those below 18 or 20 years despite documenting increased risk for those under 15 years of age.

A somewhat conflicting finding presented in one of Shemyakina’s papers [16] is that regression analysis suggested that those within the ‘war cohort’ who reached prime marriage age during the conflict and who resided in the most war-affected regions were actually less likely to be married by 18 years. This could suggest that, although at a national level marriage before aged 18 increased, particularly at the outset of the war, marriages were concentrated in the areas less directly affected by the conflict.

#### Decline in marriage

In other contexts, a decline in marriage was observed during a period of conflict [15, 18, 19, 21, 27–30]. Adverse material and economic conditions were commonly cited as key factors affecting the decline observed. For example, Saxena et al. [21] examined nuptuality trends and patterns in Lebanon during civil war. The proportion of married young women aged between 15 and 19 years declined from 13 to 5 % between 1970 and 1996. The authors concluded that the civil war made it difficult for young people to find employment and affordable housing so delayed getting married. Further, women were frequently confined to the home during periods of intense violence resulting in a decline in the opportunity to meet potential partners. Khawaja et al. [30] examined changes in marriage rates during the second Intifada using survey data from 1996 to 2005. The percentage of young women aged between 15 and 19 years who were married declined from 30.3 to 14.6 between 1995 and 2004 in Gaza, and from 21 to 12 over the same period in the West Bank, possibly as a result of the high costs of marriage.

In other cases, involuntary and sex ratio factors were cited as the main reasons for marriage postponement, and the unavailability of marriage partners either through excess mortality, migration or conscription appeared to reduce marriage rates. Woldemiceal [18, 19] speculated that the decline in Eritrea in the percentage of 15 to 19 year old women ever married from 38 to 31 between 1995 and 2002 was due to the mass mobilisation of young men to fight in the border conflict with Ethiopia. Blanc [27] supported these findings.

In Cambodia, marriage declined from its pre-war level up until the end of the Khmer Rouge regime due to excess mortality among young men [28, 29]. After the fall of the Khmer Rouge, however, both studies reported a marriage ‘boom’ among younger aged women, a result of increased competition for the remaining men. Closely related with partner unavailability, the breakdown of social networks due to conflict appears to negatively impact upon marriage rates among young women. For example, in Bosnia, the lack of social embeddedness caused by the genocide was believed to have dramatically decreased the odds of marriage during the war [15].

### Impact of conflict on fertility

Three studies reported little change in young women’s fertility during a period of conflict [15, 26, 31], three studies reported a decline [18, 19, 29, 32], five studies an overall increase in fertility [15, 22, 32–34] and one study reported differential changes among sub-groups of young women [30].

The majority of studies indicated that fertility trends were strongly influenced by the shifting trends in marriage observed among young women during time of conflict (albeit with a time lag to take account of gestation).

#### Increase in fertility

Clifford [22] reported birth rates among 15 to 19 years old women prior to, and during, the period of conflict in Tajikistan, finding there to be a steady increase in fertility among the young during the war years. Increases in fertility among young women under 20 years were also evident during the first Intifada in the West Bank and Gaza; Fargues [32] identified a sharp increase in fertility between 1985 and 1991 in both regions. Likewise, Khawaja [33] and Khawaja and Randall [34] examined fertility during the first Intifada. Between 1968 and 1991, teenage fertility increased by 700 % in Gaza and 300 % in the West Bank, with the greatest increase occurring during the Intifada period; similar increases were not, however, evident among refugee and non-refugee populations in Jordan. The authors concluded that the rise in Palestinian fertility resulted largely from a surge in marriage and, to a lesser extent, from higher marital fertility possibly due to curfews and closures leading to increased opportunity for sexual activity.

Staveteig [15] revealed that young women between 15 and 19 years at the start of the Rwandan genocide were more likely to have a first birth before they reached the age of 20 than were the two earlier cohorts (those aged between 20 and 24 and between 25 and 29 at the start of the conflict). Fertility returned to normal levels post genocide.

#### Decline in fertility

In other contexts, a decline in fertility has been observed during periods of conflict. In Bangladesh, Curlin et al. [31] identified a sharp fall in fertility among young women during the war which continued two years post-conflict. For example, the age specific fertility rates (ASFRs) among Bangladeshi young women aged 10 to 14 years declined from a high of 16.4 births per 1000 5 years before the war, to 4.6, and 5.5 per 1000 during the period of conflict and two years post-conflict, respectively. In Cambodia, marital fertility rates were also shown to fall among 15 to 19 year old women during the Khmer Rouge regime [29]; however, fertility rose rapidly after the fall of the Khmer Rouge in line with marriage rates. The authors concluded that the desire for children (by all aged women) post-conflict occurred in order to guarantee family, social and population stability and cohesion. Further, the costs of raising children remained comparatively low.

In Eritrea, fertility among 15 to 19 year olds fell during the period of conflict, and particularly among those at the younger ages [18, 19]; for example, 22 % of 17 year olds had started childbearing in 1995 compared to 8 % in 2002, and similar reductions were observed amongst 16 year olds. The observed decline in ASFRs, however, was less than among older aged women and appeared to be part of a longer term general trend in fertility reduction. The author concluded that the fall was partly due to increase in age at marriage but also due to conflict spousal separation; in 1995, 60 % of married 15 to 19 year old women resided with their husbands, whereas by 2002 this had fallen to 31 %.

Khawaja et al. [30] examined the impact of the second Intifada on fertility in Gaza and the West Bank to find that, unlike the first, ASFRs declined across the two territories between 1999 and 2003. The decline occurred among all women, but was least among those aged between 15 and 19 years. Further examination revealed that, among young women aged between 15 and 19 years with little education, fertility actually rose during the period of the second Intifada.

#### Stagnation in patterns of marriage or fertility

One article showed the immediate effect of war was to stall both declines in adolescent fertility and marriage. In 1967, the legal age of marriage was raised in Iran, a family planning programme was introduced and abortion legalised in an attempt to reduce population growth. Consequently, fertility in the country began to decline, particularly among older women, alongside increasing age at first marriage [35]. Following the revolution in 1979, Islamic republic officials stated women’s primary role was marriage and child-raising and the family planning programme was shut down. As the war with Iraq continued in the early 1980s the supply of imported goods and food declined and rationing was introduced on the basis of family size; in 1976, the ASFR was 150 births per 1000 15 to 19 years old women compared to 149 per 1000 in 1986. Although this was not a deliberate pro-natalist policy it operated as an incentive to have more children and thus caused a stalling of the pre-revolution fertility transition. It is interesting to note that a further study by the same author found the proportion of women married aged between 15 and 19 year olds during this period increased slightly in urban areas but fell in rural areas [20]. The author suggested this is because of increasing emphasis on traditional gender norms in urban areas while, in rural areas, young men migrated leading to a sex imbalance.

Randall’s [26] analyses showed that, despite an increase in early marriage during the conflict in Mali, fertility rates among 12 to 14 and 15 to 19 year olds did not appear to change. In Bosnia Herzegovina, fertility also appeared to remain relatively stable during the period of conflict [15]. After the war, however, there was a marked decline which reflected a shift in marriage patterns.

## Discussion

### Summary of findings

The 21 papers included in our study present quantitative evidence for armed conflict having an impact on marriage and births to young women under the age of 20 years at the national or sub-national level. There are examples of both increases and declines in Asia and Africa, and in the Gaza and West Bank there are actually different directions of effect for two different conflicts.

Table 3 shows a summary of the possible causal pathways through which conflict can influence marriage grouped within the three themes identified earlier: involuntary, gender and psychosocial and economic and material. We focus on marriage because changes in fertility were all associated with changes in marriage patterns. However, we recognise that this may not always be the case (e.g. fertility may rise due to births out of wedlock, or marital fertility patterns may change). We only have one study which examines marital fertility, and this suggests changes in fertility post conflict result from differing rates of coital frequency [29]. In some cases, the pathway may be quite simple; for example, marriages are postponed due to separation of partners or more pressing concerns, or increased poverty may make marriage celebrations unaffordable during conflict.

**Table 3:** Summary of suggested causal pathways relating to marriage

	Involuntary	Gender and psycho-social	Economic and material
Marriage increase	Increased opportunity for social contact between the sexes	Fear of sexual violence or loss of honour for unmarried women leads to marriage at younger ages	Disruption in girls schooling leading to earlier marriage/childbirth
	Increased competition for remaining males as marriage partners	Desire for increased social cohesion	Increased poverty leading to transactional marriages/early marriage to gain bride price
		Increased pro-natalism and conservatism supports early marriage and childbirth	
Marriage decrease	Disruption and separation of the sexes		Cost of marriage, lack of employment opportunities and destruction of infrastructure
	Lack of available males due to conscription and differential mortality		

In other cases, however, age of marriage may be manipulated to mitigate some of the potential risks and threats of conflict. This may result in changes in norms around age of marriage and birth as the underlying cultural and social practices interact with particular aspects of conflict to influence potential outcomes of marriage age. Increasing early marriage in Syria, for example, appears to have occurred as a convergence of concerns about unmarried women’s honour and safety in times of turmoil and violence alongside potential opportunities for financial benefit as a result of cultural norms around bride price for the family in times of conflict-induced poverty. Increasing early marriage during conflict tends to occur in contexts where cultural practices, norms and values already support it - although in some cases ideology fuelled by the conflict may additionally reinforce conservative attitudes (e.g. in Gaza and West Bank and Iraq); Eritrea, however, offers an exception to this general trend.

Norms of age of marriage prior to the war will affect the extent to which changes in the age of marriage will affect young people. If the norm is that most women marry between the ages of 20 and 24 years, a significant proportion of women who are this age group at the outbreak of conflict will be already married; any pathways for change will impact on those at an age ‘preparing’ for marriage; that is, those at the younger end of the 20 to 24 years age group and those under 20 years. This is a particularly important concept when considering how conflict can increase early marriages; these data highlight the importance of disaggregating data by age for adolescents in further studies.

It is also important to acknowledge that there are a number of papers which provide more general information about the impact of conflict on rates of marriage or fertility based on a wider age group. Our paper focuses on changes in patterns for those under the age of 20 years as this is the group who are most likely to experience adverse impacts from these events, so papers that did not specifically provide data on this age group were excluded. However, these excluded papers provide important contextual information that can support and extend our analysis: A study by Williams et al. [36] found an increased likelihood of marriage for women aged beween 15 and 24 years exposed to conflict in Nepal, which supports Valente’s findings of increased marriage amongst women under the age of 20 years [24]. Lindstrom and Berhanu’s [37] findings of a short term reductions in fertility in response to upheaval in Ethiopia complement Blanc and Woldemichael’s [18, 19, 27] findings on a reduction in marriage before 20 years of age in response to conflict. A study by Khlat et al. [38] found a reduction in fertility in wartime Beirut, which again aligns well with Saxena’s findings on reduced rates of marriage before 20 years of age during conflict in Lebanon [21]. Jayaraman et al. [39] suggest exposure to the Rwandan Genocide resulted in a decrease in fertility which initially seems at variance with Staveteig [15], but Jayaraman utilises more complex methods that identify levels of exposure to conflict compared to Staveteig’s analysis of temporal trends within the whole population, so the studies are not comparable. A further study by Verwimp [40] found an increase in fertility among women who became refugees as a result of the genocide, highlighting how complex patterns are likely to vary between different population groups.

### The difficulties of measuring exposure to conflict

Our review has a number of limitations rooted in methodological and practical difficulties associated with measuring exposure to conflict. Nearly all of the results in this study were based on time trends for national level data, but this does not acknowledge that some sectors of the population were much more affected by the conflict than others; Shemyakina’s work [16, 17] suggests that marriage patterns may be quite different in areas affected to greater and lesser degrees by the armed conflict. One of the two studies that attempted to address this was Valente [24], who used measures of between-district conflict intensity. Improved monitoring and reporting of conflicts through projects such as the Armed Conflict Location and Event Data (ACLED) - which provide georeferenced data of events - makes such approaches more feasible. More sophisticated approaches are emerging; for example, Williams et al. [36] examined population responses to armed conflict using an approach that decomposes the conflict in Nepal into discrete political and violent events, and estimated differential risk within the population. Jamayraman [39] measured the impact of conflict on marriage and fertility using small area level data on mortality of siblings in Rwanda. These methods still have limitations; for example, they do not deal well with internal mobility between time points.

Even when studies are able to measure exposure to conflict more accurately, there are also particular groups within populations who are more or less vulnerable to the potential effects of conflict. Most studies are at the population level which may result in these differences being lost. It is highly possible that there could be different impacts in different populations within the same country, but efforts would need to disaggregate populations to detect these.

Examination of time trends also does not enable the separation of the impact of conflict from secular trends or other contemporaneous factors. Techniques such as difference-in-difference modelling may offer some advantages in establishing a clearer association, but there are still difficulties in unravelling the impact of armed conflict from that of the wider concurrent socio-economic turbulence and uncertainty. While this paper focuses on armed conflict, other studies have found changes in fertility and nuptuality occur during other periods of non-violent turmoil and social change, and it is very challenging to establish the extent to which the presence of violence drives change. Clifford [22] presented findings of trends from four newly independent states during the turbulent period of the early post-Soviet era; while only two of the countries experienced armed conflict, all four countries demonstrated very similar trends in marriage, suggesting that caution is needed before attributing change specifically to hostilities.

### Difficulties in measuring indicators

Many of the studies rely on retrospective data from surveys or censuses which are acknowledged to have difficulties with the measurement of marriage, first births and sexual debut. Surveys such as the DHS employ a loose definition of marriage which will include legal and ‘traditional’ marriages, as well as consensual unions with cohabitation [41]. In cultures where such unions are more fluid, data quality on marriage is likely to be compromised [42]. Measures of young people’s SRH events are also likely to be subject to social desirability bias; respondents may feel the need to adjust their responses to ensure they are viewed favourably by others. In addition, in a number of studies, data from different age cohorts from a single survey were used to ascertain trends, which can be problematic as it can introduce bias, including the overstating of age [43].

### Understanding longer term outcomes

To fully understand the association between conflict and a reduction in early marriage, cohorts need to be followed up for some time after the cessation of hostilities in order to ascertain whether marriage is simply postponed to an older age group and/or these young women remain unmarried. As previously discussed, several studies that permit this suggest the decrease in marriage during conflict is followed by a marriage ‘boom’ after the end of the conflict [28, 29], a pattern supported more broadly for other studies on fertility and marital rates among post-conflict populations [44].

However, there is evidence from other studies that did not meet our specific criteria that, in some cases post-conflict, women may be less likely to marry [45] and, in cultures where marriage provide protection and status, this may be extremely damaging for a woman’s future security. It is important that post-war reconstruction efforts understand and respond to the needs of young women who may be marrying later than the societal norm or remaining single; opportunities for education and livelihood opportunities are needed to ensure their security both in the short and the longer term. Conversely, we do not understand whether increases in teen marriage and fertility experienced during conflict persist after the cessation of hostilities; given that many of the factors driving this trend (e.g. increased poverty and insecurity and loss of livelihoods) may continue into the post-war period this is highly possible. In Randall’s study [26] the marriage rate in very young girls reverted to normal quickly whereas, in Clifford’s study [22], the rate of marriage stayed elevated for some time. Again it is important to incorporate strategies into post-conflict reconstruction to protect young women who may continue to be at risk of early marriage, as well as to support those who have already been married.

### The importance of qualitative data in understanding young women’s SRH in conflict

One of the marked limitations of this review arises because its primary aim was to document *quantitative* change in rates of SRH events in women aged under 20 years; it therefore excluded qualitative studies that would have been valuable in developing insight into the pathways through which conflict can influence these outcomes. As such, we acknowledge that our analysis is incomplete; in particular, in all the studies we examined marriage and fertility are closely linked. This may not always be the case, and it is probable that, in some contexts, change in fertility may be driven by patterns of fertility outside marriage. In addition, this review cannot describe how the meaning and context of our chosen indicators changed *as a result of* conflict; for instance, Muhwezi et al. [3] described how a breakdown of community cohesion resulting from armed conflict in Uganda brought about a complete change in cultural values, with increases in transactional sex and marriage, sexual violence and high risk sexual behaviours such as multiple partners. Other studies [46, 47] document the rise of forced marriage in some contexts where women are abducted by the militia; such ‘marriages’, where there is the full consent of neither the woman nor her family, clearly differ from traditional practices, but most quantitative data sources would not be able to differentiate these from other forms of marriage. We are planning a further study which will review a broader range of literature to build on our preliminary analysis of causal pathways, as well as outline how the nature of these events may change.

## Conclusion

The aim of a systematic review is to explore the quality and quantity of research on a specific topic, while avoiding the undue influence of pre-conceived notions of what might be found. This review has highlighted a relative lack of high quality studies as well as a rather narrow range of methodological approaches and limited contexts and scope. This comment is certainly not meant as a criticism of past researchers’ efforts; high quality research in this - and similar - areas is extremely challenging, and may simply be beyond the reach of scientific endeavour using ‘gold standard’ approaches. However, we have highlighted a range of novel approaches which have been utilised by researchers to examine other demographic outcomes in conflict situations, and we suggest that these could be extended to examine early fertility and marriage.

Having said this, some tentative conclusions can be drawn based on this review. First, the effects of armed conflict on early marriage and child birth are inconsistent, and appear to vary according to a range of psychosocial, economic and conflict-related factors. Second, little work has been carried out that enables more detailed understanding of specific impacts on different groups, disaggregated, for example, by age, by rural or urban residence, by family structures, by level of poverty. Third, we came across no work that increased understanding of community- or individual-level factors or circumstances that increased resilience in the face of armed conflict.

We recommend that future research make better use of a wider range of methods to understand the dynamics of the actual and potential impact of armed conflict on young women’s sexual and reproductive health outcomes. Qualitative approaches can be designed to generate deeper understanding of patterns observed from survey analysis, and/or to generate new ways of understanding factors that are not currently captured in traditional survey items. Furthermore, the quality of survey data can be improved by strong training and supervision of fieldworkers, as well as expansion of conflict-related indicators. Ideally, some form of triangulation of data can be achieved by mixed method approaches, and greater use of penetrative methods should enable stronger conceptual clarification.

Improved understanding is urgently needed to support and guide the efforts of support and rehabilitation agencies; there is clearly much more to do.

## Additional files

Additional file 1:
**Appendix 1.** Search terms used. (DOCX 22 kb) Additional file 2:
**Appendix 2.** Prisma flowchart. (DOCX 29 kb) Additional file 3:
**Appendix 3.** Summary of sources of data and analyses used. (DOCX 37 kb) Additional file 4:
**Appendix 4.** Summary of outcome measures and key results. (DOCX 52 kb)

## Abbreviations

ASFR: age specific fertility rate
DHS: demographic and household survey
SRH: sexual and reproductive health
WFS: world fertility survey
