# Supplementary material for: The impact of armed conflict on adolescent transitions: a systematic review of quantitative research on age of sexual debut, first marriage and first birth in young women under the age of 20 years
Source: BMC Public Health. 2016 Mar 4;16:225. doi: 10.1186/s12889-016-2868-5 (PMC4779256; doi:10.1186/s12889-016-2868-5)
Supplement: Additional file 1: — Appendix 1. Search terms used. (DOCX 22 kb) [file 12889_2016_2868_MOESM1_ESM.docx]

**Appendix 1: Search terms used**

| **search terms** | | |
| --- | --- | --- |
| **population / descriptor** | **outcome** | **exposure** |
| A  teen*  young  youth  early  adolescen*  age-specific | B  pregnancy  motherhood  birth  fertility  marriage  nuptial*  cohabit*  fertility  child-bearing  conception | D  armed conflict  violent conflict  post-conflict  upheaval  fragile state  war  genocide  refugee  IDP  internally displaced people  internally displaced persons |
|  | C  child marriage  sexual debut  age at first sex  age at first birth  age at marriage |  |

Search: ((Group A AND Group B) OR Group C) AND (Group D)
