# Supplementary material for: The impact of armed conflict on adolescent transitions: a systematic review of quantitative research on age of sexual debut, first marriage and first birth in young women under the age of 20 years
Source: BMC Public Health. 2016 Mar 4;16:225. doi: 10.1186/s12889-016-2868-5 (PMC4779256; doi:10.1186/s12889-016-2868-5)
Supplement: Additional file 2: — Appendix 2. Prisma flowchart. (DOCX 29 kb) [file 12889_2016_2868_MOESM2_ESM.docx]

**Appendix 2: Prisma flowchart**

Additional records identified through other sources
(n=37)

Records identified through database searches
(n=1463)

Full-text articles assessed for eligibility
(n=51)

Records excluded
(n=1247)

Records screened
(n=1298)

Records after duplicates removed
(n=1298)

Full-text articles excluded, with reasons (n=29)

Outcomes did not meet inclusion criteria (n=9)

Conflict comparison did not meet inclusion criteria (n=11)

Neither conflict comparison nor outcome met inclusion criteria (n=4)

Source of data not given (n=1)

Paper not available (n=2)

Papers included in study (n=21)

Individual studies included

(n=19)
