# Supplementary material for: The impact of armed conflict on adolescent transitions: a systematic review of quantitative research on age of sexual debut, first marriage and first birth in young women under the age of 20 years
Source: BMC Public Health. 2016 Mar 4;16:225. doi: 10.1186/s12889-016-2868-5 (PMC4779256; doi:10.1186/s12889-016-2868-5)
Supplement: Additional file 3: — Appendix 3. Summary of sources of data and analyses used. (DOCX 37 kb) [file 12889_2016_2868_MOESM3_ESM.docx]

**Appendix 3: Summary of sources of data and analyses used**

| **Author** | **Country** | **Conflict** | **Comparison** | **Data sources** | **SRH outcome** | **Analysis** |
| --- | --- | --- | --- | --- | --- | --- |
| Aghajanian, 1991a | Iran | Islamic Revolution 1979  Iran- Iraq war 1980-88 | Temporal: Prior and during | Census 1966, 1976 & 1986  Survey of Population Growth of Iran (1974-76) | Marriage Fertility | Trend analysis |
| Aghajanian, 1991b | Iran | Islamic Revolution 1979  Iran- Iraq war 1980-88 | Temporal: Prior and during | Marriage registration  Census 1976 & 1986 | Marriage | Trend analysis |
| Blanc, 2004 | Eritrea | Border conflict with Ethiopia 1998-2001 | Temporal: Prior, during and post | DHS 1995 & 2002  Health Management Information System (HMIS) | Marriage | Trend analysis |
| Clifford, 2009 | Tajikistan | Civil war 1992-1997 | Temporal: Prior, during and post | MICS 2005  TransMONEE 2006 | Marriage Fertility | Trend analysis  Exponential proportional hazard model |
| Curlin, Chen, & Hussain, 1976 | Bangladesh | Civil war 1971 | Temporal: Prior, during and post | Matlab Health and Demographic surveillance system 1966/7 - 1972/3 | Fertility | Trend analysis |
| Fargues, 2000 | West Bank and Gaza Strip | Almost constant conflict 1949-present  Intense conflict 1987-1993, 1st Intifada 1987-1991 | Temporal: Prior, during and post | State of Israel Central Bureau of Statistics 1985 & 1991  Palestinian Central Bureau of Statistics 1995 | Fertility | Trend analysis |
| Heuveline & Poch, 2007 | Cambodia | Khmer Rouge genocide 1975-78/9 | Temporal: Prior, during and post | DHS 2000  Demographic-surveillance system data Mekong Island Population Laboratory (MIPopLab),  Census 1962 | Marriage Fertility | Trend analysis |
| Khawaja, 2000 | West Bank and Gaza Strip | Almost constant conflict 1949-present  1st Intifada 1987-91 | Temporal: Prior and during | Vital registration  Israeli Central Bureau of Statistics  Census 1967  Quarterly Labour Force Survey | Fertility | Trend analysis |
| Khawaja & Randall, 2006 | West Bank and Gaza Strip | Almost constant conflict 1949-present  1st Intifada 1987-91 | Temporal: Prior, during and post | Palestine Living Conditions Survey 1995  Jordan Living Conditions Survey 1996 | Fertility | Trend analysis |
| Khawaja, Assaf, & Jarallah, 2009 | West Bank and Gaza Strip | Almost constant conflict 1949-present  2nd Intifada 2000 | Temporal: Prior, during and post | Demographic and Health surveys carried out by Palestinian Central Bureau of Statistics 1995, 1996, 2000, 2004. | Marriage  Fertility | Trend analysis |
| Okae, 2009 | Uganda | Almost constant conflict 1987-present | By refugee status | Structured questionnaire survey, 2009 | Sexual debut | Cross sectional survey |
| Randall, 2005 | Mali | 2nd Tuareg Rebellion 1992-5 | Temporal: Prior, during and post | WFS 1981  DHS 2001 | Marriage Fertility | Trend analysis |
| Save the Children, 2014 | Syria | Arab Spring 2011/2 onwards | Temporal: Prior and during By refugee status | Official statistics  The State of the World’s Children 2013  UNICEF | Marriage | Trend analysis |
| Saxena, Kulczycki, & Jurdi, 2004 | Lebanon | Civil war 1975-76; 1982-86; 1989-90 | Temporal: Prior, during and post | Population and Housing Survey 1996  Labor Force Survey  1970  Beirut Health Survey 1984 & 1994 | Marriage | Trend analysis |
| Shemyakina, 2009 | Tajikistan | Civil war 1992-1997/8 | Temporal: Prior, during and post  By conflict intensity | Tajik Living Standards Measurement Survey 2003 | Marriage | Trend analysis  Linear probability regression |
| Shemyakina, 2013 | Tajikistan | Civil war 1992-1997/8 | Temporal: Prior, during and post  By conflict intensity | Tajik Living Standards Measurement Survey 2003 | Marriage | Trend analysis |
| Stavetig, 2011 | Bosnia Herzegovina  Rwanda | Genocide 1992-1995  Genocide 1994 | Temporal: Prior, during and post  Temporal: Prior, during and post | World Bank Living Standards Measurement Survey 2001, 2002  DHS 2000 & 2005 | Marriage Fertility  Marriage Fertility | Trend analysis  Trend analysis |
| Valente, 2011 | Nepal | Maoist insurgency 1996-2006 | By conflict intensity | DHS 2001 & 2006  Nepalese Living Standards Survey (NLSS) 2003/4 | Marriage | Trend analysis  Regression techniques adjusting for conflict intensity |
| de Walque, 2006 | Cambodia | Khmer Rouge genocide 1975-1978/9 | Temporal: Prior, during and post | DHS 2000   Labour Force Survey of Cambodia 2001 | Marriage | Trend analysis |
| Woldemicael, 2008  Woldemicael, 2010 | Eritrea | Border conflict with Ethiopia 1998-2000/1 | Temporal: Prior and post | DHS 1995 & 2002 | Marriage  Fertility | Trend analysis |

**References**

Aghajanian, A. (1991a). Population change in Iran, 1966-86: A Stalled demographic transition? Population and Development Review, 17, 703-715.

Aghajanian, A. (1991b). Women’s roles and recent marriage trends in Iran. Canadian Studies in Population, 18, 17-28.

Blanc, A. K. (2004). The role of conflict in the rapid fertility decline in Eritrea and prospects for the future. Studies in Family Planning, 353, 236-245.

Clifford, D. (2009). Marriage and fertility change in post-Soviet Tajikistan. Doctoral Thesis, School of Social Sciences, University of Southampton,, Southampton UK

Curlin, G. T., Chen, L. C., & Hussain, S. B. (1976). Demographic crisis: The impact of the Bangladesh civil war (1971) on births and deaths in a rural area of Bangladesh. Population Studies, 30, 87-105

Fargues, P. (2000). Protracted national conflict and fertility change: Palestinians and Israelis in the twentieth century. Population and Development Review, 26, 441-482

Heuveline, P. & Poch, B. (2007). The phoenix population: Demographic crisis and rebound in Cambodia. Demography, 44, 405-426.

Khawaja, M. (2000). The recent rise in Palestinian fertility: Permanent or transient? Population Studies, 54, 331-346.

Khawaja, M. & Randall, S. (2006). Intifada, Palestinian fertility and women's education. Genus, 62, 21-51.

Khawaja, M., Assaf, S., & Jarallah, Y. (2009). The transition to lower fertility in the West Bank and Gaza Strip: evidence from recent surveys. J Pop Research, 26, 153-174.

Okae P. G. (2009). A comparative study of patterns of sexual behaviour among adolescents in internally displaced people's camps and normal settlements, in Lira district. Master of Science in Population and Reproductive Health, Makerere University, Uganda.

Randall, S. (2005). The demographic consequences of conflict, exile and repatriation: A case study of Malian Tuareg. Eur J Population, 21, 291-320.

Save the Children (2014). Too Young to Wed: The growing problem of child mariage among Syrian girls in Jordan. London: Save the Children.

Saxena, P., Kulczycki, A., & Jurdi, R. (2004). Nuptiality transition and marriage squeeze in Lebanon: Consequences of sixteen years of civil war. Journal of Comparative Family Studies, 35, 241-258.

Shemyakina, O. (2009). The marriage market and Tajik armed conflict ( Health in Conflict Network Working Paper 66). University of Sussex: The Institute of Development Studies.

Shemyakina, O. (2013). Patterns in female age at first marriage and Tajik armed conflict. Eur J Population, 29, 303-343.

Staveteig, S. (2011). Genocide, Nuptiality, and Fertility in Rwanda and Bosnia-Herzegovina. Doctoral thesis, Sociology and Demography, University of California, Berkeley.

Valente, C. (2011). What Did the Maoists Ever Do for Us? Education and Marriage of Women Exposed to Civil Conflict in Nepal. Policy Research Working paper 5741, Washington DC: The World Bank.

de Walque, D. (2006). The socio-demographic legacy of the Khmer Rouge period in Cambodia. Population Studies, 60, 223-231.

Woldemicael, G. (2008). Recent fertility decline in Eritrea: Is it a conflict-led transition? Demographic Research, 18, 27-58.

Woldemicael, G. (2010). Declining Fertility in Eritrea Since the Mid-1990s: A Demographic Response to Military Conflict. International Journal of Conflict and Violence, 4, 149-168.
