# Supplementary material for: The impact of armed conflict on adolescent transitions: a systematic review of quantitative research on age of sexual debut, first marriage and first birth in young women under the age of 20 years
Source: BMC Public Health. 2016 Mar 4;16:225. doi: 10.1186/s12889-016-2868-5 (PMC4779256; doi:10.1186/s12889-016-2868-5)
Supplement: Additional file 4: — Appendix 4. Summary of outcome measures and key results. (DOCX 52 kb) [file 12889_2016_2868_MOESM4_ESM.docx]

**Appendix 4: Summary of outcome measures and key results**

| **Short Title** | **Country** | **Measure** | **Findings** | **Suggested causal pathway** |
| --- | --- | --- | --- | --- |
| Aghajanian, 1991a | Iran | Proportion of women ever married, 15-19 years | The percentages of women ever married aged 15-19 years declined between ’66 and ’76 and then plateaued during the next decade.  1966 - 46.5  1976 - 34.2  1986 - 33.5  The percentages of women currently married aged 15-19  Iran Urban Rural  1966 44.8 38.9 48.9  1976 33.9 30.2 37.6  1986 33.2 31.8 34.0 | *1966-1976*: Increasing liberalism and gender equality (including female education)  *1976-1986*: Post revolution - decline in legal age of marriage, increasing conservatism and pro-natalism and strengthening gender norms relegating women to traditional roles |
|  |  | Age specific fertility rates, 15-19 year olds | Between 1966 and 1986 fertility among over 20s declined but remained static among the under 20s  ASFRs per 1000 women  1966 1976 1986  15-19 45^[[1]](#footnote-1)^ 150 149  20-24 375 316 303 | Associated with changes in marriage  1966-76 onset of fertility decline, increasing use of contraception  Food insecurity and rationing |
| Aghajanian, 1991b | Iran | Proportion of women ever married, 15-19 years  Number of 10-14 year olds married | Between ’76 and ’86 the percentages of women ever married aged 15-19 years increased slightly in urban areas but fell in rural areas  Iran Urban Rural  1976 34.2 30.5 38.1  1986 33.5 32.5 34.1  In the 1986 census 31,577 women aged 10-14 were reported as married, compared to 500 fewer in 1976 | *Urban areas:* Traditional gender norms, increasing conservatism and pro-natalism  *Rural areas:* Marriage squeeze due to young male migration in search of better opportunities  Financial and social incentives for early marriage were offered |
| Blanc, 2004 | Eritrea | Proportion of women ever married, 15-19 year olds | The percentages of women ever married aged 15-19 years declined during the conflict  1995 2002  15-19 37.6 31.0 | Mobilisation of men due to conflict |
| Clifford, 2009 | Tajikistan | Hazard ratio of marriage  Relative risk of union formation, 15-17 and 18-20 year olds | The relative risk of marriage for young girls aged 15-17 increased from its baseline in 1988 (prior to end of Soviet rule) to reach a peak of 2.36 in 1994 (during the conflict). A decline followed although the relative risk did not return to around 1 until 2000  Among 18-20 year olds (prime marriage ages) risk of marriage increased to a high of 1.3 in 1991/2 then declined steeply during the early conflict years (1993-95), and consistently fell thereafter | *During the transitional years:*  Increased post-soviet conspicuous consumption and wealth;  Increased nationalism leading to resurgence of early marriage;  Concerns about insecurity;  Perceived need to protect honour of young women.  *Post transition and during conflict:*  Food insecurity reduced marriage |
|  |  | Birth rates, 15-19 year olds  Relative risk of first birth | Steady increase in birth rates from ’89-95 (dip in ’92 when war broke out)  Sharp decline in relative risk of first birth in 1996 | Associated with changes in marriage  Food insecurity and malnutrition |
| Curlin, Chen, & Hussain, 1976 | Bangladesh | Age specific fertility rates, 10-14 and 15-19 year olds | Overall fertility (all aged women) was relatively stable during and after the conflict. However, there was a sharp fall in fertility among younger women (10-14 and 15-19) during, and in the two years following, the conflict.  ASFRs per 1000 women  Av 5yrs before war War 1971/2 1972/3  10-14: 16.4 4.6 5.5  15-19: 216.0 192.0 139.0 | Associated with changes in marriage patterns due to conflict |
| Fargues, 2000 | West Bank and Gaza Strip | Age specific fertility rates, 15-19 year olds | Fertility in women aged 15-19 increased sharply during the intifada. It appears to then reduce slightly afterwards  ASFRs per 1000 women  1985 1991 1995  West Bank 73 160 100  Gaza 79 195 145 | Associated with changes in marriage and a reduction in cost of marriage (bride price)  Social conditions encouraged fertility  Increased pro-natalist policies and ideologies |
| Heuveline & Poch, 2007 | Cambodia | Age specific marriage rates (ASMR), 15-19 year olds | ASMRs among 15-19 year olds fell significantly from their pre-war level during the Khmer Rouge (KR) regime. Following the KR fall there was a marriage boom (doubling the pre-war rate) but marriage rates returned quickly to the pre-war level | Disproportionate male mortality  Marriage boom more than just a catch up for delayed marriages. Increased competition for remaining males forced young women to marry quickly (even to take men who previously had been deemed unsuitable)  Lack of male partners and its constraint on the marriage market lowered marriage rates after 1980 |
|  |  | Age specific marital fertility rates, 15-19 year olds | Fertility fell during the KR regime for all age groups including those who were under 20 years at the time. After the fall of the KR it rebounded rapidly among women of all ages. Women who were around age 15 at the time of the KR takeover contributed to the post-KR marital fertility surge, even though they most likely did not have their reproductive career interrupted | Associated with changes in marriage  *Post-conflict fertility boom related to:*  guaranteed robust family and social cohesion  costs of raising children remained comparatively low  other forms of investment (e.g., livestock and machinery) had been made scarce by the KR regime  potential higher coital frequency |
| Khawaja, 2000 | West Bank and Gaza | Percentage change in age specific fertility rates, 15-19 year olds from 1968 baseline | Teenage fertility (aged 15-19) increased considerably in both Gaza (by up to 700%) and the West Bank (by 300%) during the period 1968 to 1991. Largest increases occurred during the 1^st^ Intifada period | Associated with changes in marriage  Higher coital frequency (possibly due to curfews and closures) |
| Khawaja & Randall, 2006 | West Bank and Gaza | Ratio of age specific fertility rates before and during the 1^st^ intifada, 15-19 year olds | There was an overall increase in fertility during the 1^st^ Intifada in Gaza and the West Bank (but not among refugee and non-refugee populations living in Jordan), and most marked in the 15-19 age group | Associated with changes in marriage  Reduction in age at marriage due to  increasing conservatism  school closures |
| Khawaja, Assaf, & Jarallah, 2009 | West Bank and Gaza | Proportion of young women never married, 15-19 year olds | The percentage of young women never married aged 15-19 increased between 1995 and 2004  Gaza West Bank  1995 69.8 78.8  2000 80.0 82.6 (2^nd^ Intifada)  2004 85.4 88.2 | During the 2^nd^ Intifada, marriage did not become less costly.   - Women started to delay marriage |
|  |  | Age specific fertility rates, 15-19 year olds | 1999-2003, fertility declined across the two territories, but the decline was least for the younger two groups (15-19 and 20-24)  In Gaza the declines in ASFR stagnated after the intifada (2000) in all age groups including those aged 15-19  For women aged 15-19 with only elementary education the ASFR rose sharply between 1999-2003: from 167 to 230 births per 1000 | Associated with changes in marriage |
| Okae, 2009 | Uganda | Proportion having first sex before ages 15 and 18 | 9.8% and 37.4% of young women in camp had had sex before aged 15 and aged 18 respectively compared to 7.6% and 39.0% of women in the surrounding villages. | The issue of transactional sex is mentioned, but no direct causal relationship suggested |
| Randall, 2005 | Mali | Percentage distribution of age at first marriage | During the conflict the proportion of first marriages involving very young girls (under 15) and older women (aged 19+) increased. Levels reverted to the pre-conflict distribution after the end of the conflict | Pro-natalist strategy of moving young women into reproductive situations  Large refugee camps allowed for easier matching of couples.  Provided protection for women (against premarital pregnancy)  Enabled alliances to be secured and bonds to be reinforced |
|  |  | Age specific fertility rates, 12-14 and 15-19 year olds | Little change in fertility during the 20 year period |  |
| Save the Children, 2014 | Syria | Proportion of young women married under 18 | In Jordan, the proportion of registered marriages among the Syrian refugee community among under 18 year olds rose from 12% in 2011 (roughly the same as the figure in pre-war Syria – 13%) to 18% in 2012, and 25% by 2013 | Refugees reliant on dwindling resources and lacking economic (employment and livelihood) opportunities  Protection against threat of sexual violence and other kinds of hardship  To protect family honour  To secure sponsorship to allow a girl and her family to move out of camps  To enable males to be allowed to enter other countries (more likely if men are married and have a family) |
| Saxena, Kulczycki, & Jurdi, 2004 | Lebanon | Proportion of women never married, 10-14 and 15-19 year olds | The percentages of young women never married increased between 1970 and 1996.  single females (all Lebanon)  1970 1996  10-14 - 99.9  15-19 86.8 95.0  20-24 50.9 71.9 | Delayed marriage due to adverse economic conditions, civil war made it difficult for young people to find employment and affordable housing  Increased male mortality and migration from areas more affected by conflict. Women more likely to be confined to home during periods of violence  Young females sought more education putting a squeeze on their marriage prospects |
| Shemyakina, 2009  Shemyakina, 2013 | Tajikistan | Proportion of women married by age 18 or below.  Probability of marriage by age 18. (Shemyakina, 2009)  Risk of entry into marriage by birth cohort  (Shemyakina, 2013) | The proportion of women married by age 18 or below increased by more than six percentage points for the birth cohort that reached age 18 between 1993 and 1995: the first years of the Tajik civil war.  Increases were also seen in this cohort for marriages <16 years and <17 years. However, no increase was seen for marriages by age 20 or below for the cohorts of adolescent age during the time of the conflict; instead a gradual decline in seen  Residing in the conflict affected region before age 12, reduced the probability of being married by age 18 by 6.7%  The conflict had the largest negative effect on the risk of being married for the youngest cohort that turned 15-17 towards the end of the conflict | During the war high levels of kidnap and rape of young girls. Spike in early marriages at start of war as parents rushed to marry their daughters and  transfer responsibility for the safety of girls to their new families  Delayed marriage due to adverse economic conditions – unable to pay for marriage expenses  General state of insecurity prevented Tajik households being able to project their future  Marriage delayed for migrants due to a breakdown of social networks |
| Stavetig, 2011 | Rwanda  Bosnia | Proportion of women remaining single after age 15 by age group at the start of the genocide | The birth cohort that was aged 15-19 at the time of the genocide had a similar or slightly higher likelihood of marriage before the age of 20 than earlier cohorts. Among women aged 10-14 at the time of the genocide their likelihood was reduced.  Proportional differences in delayed marriage at each age across generations. Cohort aged 15-19 at the time of the genocide were less likely to marry by the age of 20 than previous cohorts. | Young women were entering the marriage market earlier when the ‘supply’ of men was dramatically reduced  Possible increase in transactional marriage, following orphan-hood and destruction of homes and livelihoods  Delay effect due to material hardship and a lack of social embeddedness |
|  | Rwanda  Bosnia | Proportion of women remaining childless after15 by age group at the start of the genocide | The cohort who were 15-19 at start of genocide were more likely to have a first birth before aged 20 than the 2 previous cohorts. This then drops for the cohort who were 10-14 at time of genocide (similar to cohorts 20-24 and 25-29)  Fertility fairly stable during war. Cohorts aged 10-14 and 15-19 at start of the war did not show any marked difference in births before aged 20 | Associated with changes in marriage |
| Valente, 2011 | Nepal | Probability of marriage by age 15, 18 and 21 | Exposure to increased intensity of conflict (as measured by abductions) is associated with an increased probability of marriage before 15 years, but not before 18 or 21 | Possible link to parental concern over abduction |
| de Walque, 2006 | Cambodia | Probability of first marriage occurring in any specific year, by birth cohort | Women under the age of 20 years during the time of the Khmer Rouge (KR) were markedly less likely to marry than cohorts who reached the same age before or after this period. This reflected a more general fall in marriage during the KR period, which was followed by a ‘boom’ in all age groups after the KR fell. The probability of marriage for those aged around 15-19 and those aged 20-24 peaked at the same time, and patterns suggest those age 20-24 after the fall of the KR had delayed marriage | Women delayed their marriages during the conflict period. Young men victims of the excess mortality, resulting in a significant shortage of young men in the marriage market after the KR fall. To adapt, the age difference between partners reduced |
| Woldemicael, 2008  Woldemicael, 2010 | Eritrea | Proportion of women ever married, 15-19 year olds | The proportion of ever married 15-19 year olds fell from 37.6% in 1995 to 31.0% in 2002 | Conflict leading to marriage postponement  Mass mobilisation of young men may have reduced marriage opportunities |
|  |  | Age specific fertility rates, 15-19 year olds  Woldemicael (2008)  Proportion of women aged 15-19 who have begun childbearing  Woldemicael (2010) | Fertility among 15-19 year olds fell, but this drop was less than the decline among women in older age groups.  Percentage of adolescent women who have begun childbearing:  Age 1995 2002  15 3.0 2.1  16 13.4 2.8  17 21.6 8.0  18 40.4 24.0  19 50.7 36.4  15-19 23.3 14.0  Rural 33.8 19.3  Urban 7.4 7.7  No educ 42.1 25.4  1^o^ educ 18.9 13.5  2^o^+ educ 4.6 6.6  Early fertility fell between 1995 and 2002, particularly among the younger ages. Urban teenagers were less likely to experience motherhood than rural teenagers  Fertility did not fall in urban areas or among the richest | Spousal separation - many young men were conscribed  Part of a general fertility decline among all ages |

**References**

Aghajanian, A. (1991a). Population change in Iran, 1966-86: A Stalled demographic transition? Population and Development Review, 17, 703-715.

Aghajanian, A. (1991b). Women’s roles and recent marriage trends in Iran. Canadian Studies in Population, 18, 17-28.

Blanc, A. K. (2004). The role of conflict in the rapid fertility decline in Eritrea and prospects for the future. Studies in Family Planning, 353, 236-245.

Clifford, D. (2009). Marriage and fertility change in post-Soviet Tajikistan. Doctoral Thesis, School of Social Sciences, University of Southampton,, Southampton UK

Curlin, G. T., Chen, L. C., & Hussain, S. B. (1976). Demographic crisis: The impact of the Bangladesh civil war (1971) on births and deaths in a rural area of Bangladesh. Population Studies, 30, 87-105

Fargues, P. (2000). Protracted national conflict and fertility change: Palestinians and Israelis in the twentieth century. Population and Development Review, 26, 441-482

Heuveline, P. & Poch, B. (2007). The phoenix population: Demographic crisis and rebound in Cambodia. Demography, 44, 405-426.

Khawaja, M. (2000). The recent rise in Palestinian fertility: Permanent or transient? Population Studies, 54, 331-346.

Khawaja, M. & Randall, S. (2006). Intifada, Palestinian fertility and women's education. Genus, 62, 21-51.

Khawaja, M., Assaf, S., & Jarallah, Y. (2009). The transition to lower fertility in the West Bank and Gaza Strip: evidence from recent surveys. J Pop Research, 26, 153-174.

Okae P. G. (2009). A comparative study of patterns of sexual behaviour among adolescents in internally displaced people's camps and normal settlements, in Lira district. Master of Science in Population and Reproductive Health, Makerere University, Uganda.

Randall, S. (2005). The demographic consequences of conflict, exile and repatriation: A case study of Malian Tuareg. Eur J Population, 21, 291-320.

Save the Children (2014). Too Young to Wed: The growing problem of child mariage among Syrian girls in Jordan. London: Save the Children.

Saxena, P., Kulczycki, A., & Jurdi, R. (2004). Nuptiality transition and marriage squeeze in Lebanon: Consequences of sixteen years of civil war. Journal of Comparative Family Studies, 35, 241-258.

Shemyakina, O. (2009). The marriage market and Tajik armed conflict ( Health in Conflict Network Working Paper 66). University of Sussex: The Institute of Development Studies.

Shemyakina, O. (2013). Patterns in female age at first marriage and Tajik armed conflict. Eur J Population, 29, 303-343.

Staveteig, S. (2011). Genocide, Nuptiality, and Fertility in Rwanda and Bosnia-Herzegovina. Doctoral thesis, Sociology and Demography, University of California, Berkeley.

Valente, C. (2011). What Did the Maoists Ever Do for Us? Education and Marriage of Women Exposed to Civil Conflict in Nepal. Policy Research Working paper 5741, Washington DC: The World Bank.

de Walque, D. (2006). The socio-demographic legacy of the Khmer Rouge period in Cambodia. Population Studies, 60, 223-231.

Woldemicael, G. (2008). Recent fertility decline in Eritrea: Is it a conflict-led transition? Demographic Research, 18, 27-58.

Woldemicael, G. (2010). Declining Fertility in Eritrea Since the Mid-1990s: A Demographic Response to Military Conflict. International Journal of Conflict and Violence, 4, 149-168.

1. This figure is as presented in the paper, but the authors of this paper query whether it may be an error. It is not commented on in the original article. [↑](#footnote-ref-1)
